# Annotation of Human Exome Gene Variants with Consensus Pathogenicity

**DOI:** 10.3390/genes11091076

**Published:** 2020-09-14

**Authors:** Victor Jaravine, James Balmford, Patrick Metzger, Melanie Boerries, Harald Binder, Martin Boeker

**Affiliations:** 1 Institute of Medical Biometry and Statistics, Faculty of Medicine and Medical Center, University of Freiburg, 79104 Freiburg im Breisgau, Germany; balmford@imbi.uni-freiburg.de (J.B.); binderh@imbi.uni-freiburg.de (H.B.); martin.boeker@imbi.uni-freiburg.de (M.B.); 2 Institute of Medical Bioinformatics and Systems Medicine, Medical Center—University of Freiburg, Faculty of Medicine, University of Freiburg, 79110 Freiburg im Breisgau, Germany; patrick.metzger@mol-med.uni-freiburg.de (P.M.); melanie.boerries@uniklinik-freiburg.de (M.B.)

**Keywords:** variant of unknown significance (VUS), single-nucleotide variant (SNV), variant effect prediction (VEP), hit ratio (HR), stacked ensemble of supervised learners (SESL), next generation sequencing (NGS), alternative allele frequency (AAF)

## Abstract

A novel approach is developed to address the challenge of annotating with phenotypic effects those exome variants for which relevant empirical data are lacking or minimal. The predictive annotation method is implemented as a stacked ensemble of supervised base-learners, including distributed random forest and gradient boosting machines. Ensemble models were trained and cross-validated on evidence-based categorical variant effect annotations from the ClinVar database, and were applied to 84 million non-synonymous single nucleotide variants (SNVs). The consensus model combined 39 functional mutation impacts, cross-species conservation score, and gene indispensability score. The indispensability score, accounting for differences in variant pathogenicities including in essential and mutation-tolerant genes, considerably improved the predictions. The consensus combination is consistent with as many input scores as possible while minimizing false predictions. The input scores are ranked based on their ability to predict effects. The score rankings and categorical phenotypic variant effect predictions are aimed for direct use in clinical and biological applications to prioritize human exome variants and mutations.

## 1. Introduction

Accurate and exhaustive annotation of human gene variants is important for every application of NGS technology, including development of therapies, selection of an effective individualized therapy, and comparing multiple samples in biological/clinical studies. Currently, according to Genetests [1], more than 68,000 genetic tests are offered in clinics, resulting in an equally large and rapidly growing amount of experimentally sequenced data that requires analysis and interpretation. Among the many types of annotations that can be assigned to each sequenced NGS variant, of particular interest are those predicting if a variant has a benign or deleterious phenotypic effect. However, causative relationships between genes and phenotypes are very complex, depending on many connections and influences, and there are major challenges in obtaining the associations [2]. In the following paragraphs, we briefly overview the state-of-the-art approaches in annotating with phenotypic effects, focusing mostly on an array of predicted pathogenicity scores, and on population allele frequencies, as it is also very popular for estimation of pathogenicity. We list their main limitations and describe an approach proposed in this work with an eye to solving those limitations, followed by a brief overview of the method’s results.

In total, almost 100 million single nucleotide variants (SNV) are possible in the human gene-coding ‘exome’ space, of which around 83 million are non-synonymous nsSNVs and around 15 million are short splice-site ssSNVs. About 6 million SNVs (or ~6% of total) have been annotated with alternative allele frequency (AAF), obtained through large population sequencing projects [3] such as ExAC [4] and gnomAD [5]. The prime limitation of using AAF, apart from the known issue of strong population bias, is that in practice it is intrinsically difficult to choose where to assign a frequency threshold for discriminating between benign and pathogenic variants; the histogram of AAF values follows a very steep (γ) distribution, with 85% of all sequenced variants being singletons or ultra-rare. Applying a single threshold for all genes (e.g., 0.1%) does not achieve clear-cut separation. The recently-proposed approach [6] of using a different threshold for each gene can partially alleviate this problem, but the sub-distributions in genes are of the same type as the combined distribution.

Another source of annotations is evidence on mutation effects from clinical and biological studies, but currently, the coverage of the variant space by such studies is very limited. For example, ClinVar [7] annotations cover ca. 0.5% of the total possible variation. For the remaining majority, the best extant alternative is to use annotations from various variant effect prediction (VEP) tools.

The first group of VEP methods (e.g., GERP++, SiPhy, bStatistic) [8] is based on nucleotide conservation in alignments of genetic sequences in mammalian species. It uses the statistical property of rare variants being typically less evolutionarily conserved and having higher predicted pathogenicity than frequent ones [9]. The second group of VEP methods predicts the functional impact of mutations (e.g., MutationTaster, Eigen, fitCons) [9]. A third group are approaches based on structural information from protein 3D structures (e.g., Polyphen, SuSPect, SNAP2) [10]. A fourth group of ensemble methods (e.g., REVEL [9], CADD [11]) uses combinations of predictive scores of several types. Notably, the tools from the last group have shown better predictive performance than any from the first three groups [12]. For example, in a study [13] using The Cancer Genome Atlas (TCGA), a dataset comprised of 10,000 tumor samples across 33 cancer types, it was shown that improved prediction results were obtained by combining scores of all types, where the results were confirmed in 78% of cases by in vitro functional tests. When the methods in these four VEP groups are compared [14], it seems the main limitation is that even for the best tools, errors of more than 10% are common [15], which goes alongside low specificity in the range of 25–83% [16,17].

It is interesting to note that nearly all these methods provide for each variant a single continuous score value, which is usually converted to range from 0 (benign) to 1 (deleterious). A high-value continuous score for a variant implies a higher probability of it having a deleterious phenotypic effect; thus, such scores do not provide directly interpretable annotation, which is usually preferred for use in practice. Deriving a binary annotation from such score (i.e., to differentiate pathogenic or benign) requires applying a threshold in the range from 0 to 1 (e.g., 0.5). However, the common practice of using a single threshold for all genes is questionable, as the pathogenicity score values for both pathogenic and benign variants are typically very diffusely distributed over the whole 0–1 range, as can be seen e.g., in the results presented in this work. In reality, the situation is further complicated by the need to obtain thresholds for each individual gene [6]; for example, variants in essential genes typically require a lower threshold compared to those in mutation-tolerant genes. To remedy this issue, we use in training here a gene indispensability score [18] to account for differences due to highly varying degrees of gene mutation-tolerance or involvement in a number of protein-protein interactions or cellular networks [18]. Another known limitation is that there is a very large degree of contradiction between them [19], making it very difficult to derive a consensus annotation from a long list of scores for a variant—i.e., without using a very advanced computational technique, as is done here.

In contrast to the continuous scores produced by a large majority of existing methods, categorical classes such as ‘Pathogenic’, ‘Likely_benign’, or ‘Uncertain_significance’ are easier to interpret and can be used directly, i.e., without the need for any threshold determination. To the best of our knowledge, so far, no study has developed a method able to classify variants by categorical effect annotations across the entire coding genome and with sufficiently high accuracy.

The method of consensus variant effect prediction (or cVEP) proposed in this work attempts to obtain annotations of gene variants by determining a consensus of the 39 impact prediction scores compiled in dbNSFP [20]. The inclusion of scores in the database by its authors was based on comparison of their performance in a competition involving a large number of scores in a series of test cases for predicting variant impacts for new data not yet included in the ClinVar database. Thus, this selection of scores is unbiased, merit-based, and represents the current state-of-the-art in the field. Although each of the scores are based on their own respective knowledge domains, such as cross-species sequence conservation or functional impact of mutations, there often exists considerable overlap between scores if any part of data they use for training is obtained from the same source. It is very difficult to determine degrees of overlap from the associated publications; however, novel machine learning algorithms can effectively address the problem of redundancy in training information, as well as other notorious problems such as over- and under-fitting of input data in models, or highly unbalanced datasets. For example, random forest (RF) algorithms such as distributed random forest (DRF) [21], and gradient boosting machines (GBMs) [22] are known to deal well with redundancy by being specially designed for related techniques, such as determining feature importance, which evaluates the degree of unique contribution from each score. The approach used here is a stacked ensemble of base-learners. Described simply: a Generalized Linear Model (GLM) determines which features are the most non-contradictory to the target of prediction; a GBM determines the largest unique and orthogonal contributions; and a DRF takes all contributions from features that are consistently in line with the target. Additionally, since there remains a lack of consensus regarding which method is best for predicting the effect annotation, we provide a ranking of the methods by their contribution to correct predictions.

A Stacked Ensemble of Supervised Learners (SESL) is a two-level machine learning algorithm that combines several prediction algorithms, or base-learners, using a process called stacking (also known as meta- or super-learning). Unlike bagging and boosting approaches, which are also used for combining base-learners, the goal in stacking is to combine a (preferably diverse) set of learners together. It has been shown [23] that a super-learner ensemble represents an asymptotically optimal system for learning. Among stacking algorithms, super-learning is distinguished by the direct implementation of cross-validation and for tuning parameter selection in the algorithm. The method classified variants into five putative annotation categories: pathogenic, likely pathogenic, uncertain significance, likely benign, benign. This classification is in accordance with the standards and guidelines of the American College of Medical Genetics and Genomics (ACMG) and of the Association of Molecular Pathology (AMP) [24]. The predicted class annotations are trained and cross-validated on multiple categories of ClinVar data, which are rather accurate, since the ACMG/AMP expanded guideline for inclusion requires each variant classification to have certainty of better than 90% from experimental clinical/biological evidence [25].

To summarize the results of the study: first, we illustrate how variant class discrimination improves with increasing dimensions of input data. Then, we describe a meta-learning consensus method that combines a selection of the best-performing functional impact effects and sequence conservation and pathogenicity scores, along with one gene-level score. We then train and cross-validate the consensus ensemble meta-model on a dataset of known effect annotations derived from the ClinVar database. We provide rankings for the ability of these scores to make correct predictions, both individually and in combination, derived from feature importance in the base models. We apply the ensemble model to predict effect annotation classes for human exonic variants in all chromosomes. Finally, to demonstrate the potential of the method for use in applications, we provide data on applying it to several highly pathogenic genes.

## 2. Materials and Methods

### 2.1. Machine Learning Algorithm

To perform SESL we use the H2O tool [26], which is an open source, in-memory, distributed, fast, scalable machine learning and predictive analytics tool that allows users to build machine learning models for big data analysis, implemented as a package in the statistical environment R. The ‘meta-learning’ algorithm, implemented here using the *h2o.stackedEnsemble* function of the H2O tool (version 3.0), finds an optimal combination of the following three base learners: generalized linear model (GLM) [27], gradient boosting machine (GBM) [22], and distributed random forest (DRF) [21].

The popular GLM method is a well-known generalization of linear regression for response variables. More recently developed DRF and GBM methods both build collections of decision trees. A decision tree captures interactions among different features, with the interactions order controlled by a tree depth parameter–higher order for higher depths. There are significant differences in these algorithms: briefly, DRF averages multiple decision trees, created on different random samples of rows and columns; GBM builds the model in a stage-wise fashion. The algorithms are non-linear, robust for noisy data, and provide importance of each predictor in the models. Here, the base-models use ‘multinomial’ distribution for class prediction; the types of distributions used for continuous combination are given in Supplementary Materials.

The key parameters (*max_depth*, *ntrees*, *max_after_balance_size*, *learn_rate/sample_rate*, *min_rows*) for DRF, GBM, and GLM were chosen using the grid search in the parameter space to produce fits with the lowest logarithmic score and with its achieved stability in the five-fold cross-validations. The logarithmic score [28] method is connected to Shannon entropy and Kullback–Leibler divergence, denoted by the term *logloss* (Equation (1)) in H2O documentation. It measures the performance of a multinomial classification model during minimization. *Logloss* for a perfect model would be equal to 0, and it increases as the predicted probability diverges from the actual label. For the multi-class classification, the sum of *logloss* values for each class prediction in the observation is taken:

*Logloss* = −Σ*_c_ y_c,o_ log*(*p_c,o_*) (1)

where *y_c_*_,*o*_ is a binary indicator (0 or 1) of whether class label *c* is the correct classification for observation *o*; *p_c_*_,*o*_—the model’s predicted probability that observation *o* is of class *c*.

Since there were a different number of points for each class, the learning was performed with balancing classes. The meta-learner used GLM with nonnegative weights. When the algorithms were learning categorical values, instead of the R^2^, the McFadden’s pseudo R-squared, or likelihood-ratio index, was used. It is a statistical measure of closeness of the predicted and input data are in the fits. The log likelihood of the intercept model is treated as a total sum of squares, and the log likelihood of the full model is treated as the sum of squared errors. The ratio of the likelihoods scaled to 0–1 range gives the level of improvement offered by the full model over the intercept model. When comparing two models, McFadden’s ratio is higher for the model with the greater likelihood.

### 2.2. Datasets

The gnomAD [5] dataset extends the ExAC [4] dataset from 60,706 to 125,748 unrelated individuals of a diverse set of ethnicities, including European, African, Latino, South Asian, East Asian. It contains allele frequencies of the sequenced genetic (mostly exome) variants. ClinVar [7] is a public archive of the relationships of human variations and phenotypes, with supporting evidence. Its interpretations of the clinical significance of germline and somatic variants for reported conditions are based on “ground-truth”-clinical and biological evidence. The archive located at the National Center for Biotechnology Information (NCBI) is maintained according to standards and guidelines of the American College of Medical Genetic and Genomics (ACMG) and the Association for Molecular Pathology (AMP) [25]. Here, the ‘ClinVar dataset’ is the subset of the gnomAD dataset of 227,232 nsSNVs having non-empty ClinVar annotations. The dbNSFP [20] is a database integrating annotations from multiple sources, including allele frequencies from ExAC, gnomAD, clinical annotations from ClinVar, and functional and conservation annotations [20]. Its current academic version (“a”-branch) compiled prediction scores for 84,013,490 non-synonymous SNVs (nsSNVs), from 29 functional prediction algorithms (*SIFT*, *SIFT4G*, *Polyphen2-HDIV*, *Polyphen2-HVAR*, *LRT*, *MutationTaster2*, *MutationAssessor*, *FATHMM*, *MetaSVM*, *MetaLR*, *CADD*, *VEST4*, *PROVEAN*, *FATHMM-MKL coding*, *FATHMM-XF coding*, *fitCons*, *LINSIGHT*, *DANN*, *GenoCanyon*, *Eigen*, *Eigen-PC*, *M-CAP*, *REVEL*, *MutPred*, *MVP*, *MPC*, *PrimateAI*, *GEOGEN2*, *ALoFT*), 9 conservation scores (*bStatistic*, *phyloP100way_vertebrate*, *phyloP30way_mammal*, *phyloP17way_primate*, *phastCons100way_vertebrate*, *phastCons30way_mammal*, *phastCons17way_primate*, *GERP++ and SiPhy*) and other annotations (Table S1).

Each of the curated training, prediction, and validation datasets contained the following annotations from the dbNSFP4.0 [20]: 39 functional annotation scores (Table 1, the sources are listed in Table S1); the gene-level *Gene_indispensability* score, a probability prediction of whether the gene is essential (from 0 to 1) [18]; and two pairs of AAF fields for the exome and control samples: *gnomAD_exomes_AF* of all exome samples (125,748 samples); *gnomAD_exomes_POPMAX_AF* is the maximum across populations; *gnomAD_exomes_controls_AF* is for the ‘controls’ subset (54,704 samples; *gnomAD_exomes_controls_POPMAX_AF* is the maximum in the ‘controls’ subset.

The *Gene_indispensability* score was included due to its adding a significant improvement in predictive performance, particularly for rare variants and VUSs. It is well known that a moderately deleterious variant in a non-essential gene can often exhibit itself as non-pathogenic, as e.g., such can be located in a mutation-tolerant gene, or in a truncated dis-functional gene having a duplicate functional gene; whereas such a variant in an essential gene is usually very pathogenic, since such genes are involved in several cellular networks, leading to a major cell function disruption. The original model [18] derives an ‘*Indispensability*’ score from the degree-centralities and other characteristics in a ‘multinet’ of the networks: protein-protein interactions (PPI), phosphorylation, signaling, metabolic, genetic, regulatory, essentiality, LoF-tolerance, number of PPI interfaces, dN/dS ratios, paralogs, etc. The score value is higher e.g., for variants in oncogenes and tumor suppressors, while it is near 0 in duplicated and mutation-tolerant genes.

### 2.3. Training, Prediction, and Validation Procedures

The 40 scores (Table 1) and the indispensability were derived from the dbNSFP. Since the *LINSIGHT* (x31) score was missing for most of the gnomAD variants, it was replaced with a ‘Mean’ of 39 scores available for each variant (excl. indispensability).

The combined “gnomAD-AAF” dataset consisted of 5,502,403 variants having *gnomAD_exome_AF* annotations. This dataset was further subdivided into ‘Control’, with non-zero *gnomAD_exome_control_AF* annotations (3,451,486 variants for 54,704 samples from individuals not associated with any disease), and ‘Disease’, which included the remaining 2,050,917 variants (i.e., annotated with *gnomAD_exome_AF* but not with *gnomAD_exome_control_AF*). There were two AAF versions used: *gnomAD_exome_AF* and *gnomAD_exome_AFpopmax*.

All nsSNV variants listed in the dbNSFP4.0 were classified into the categories by the ClinVar annotations. The ‘training’ (and cross-validation) dataset comprised the variants with ClinVar annotations (133,514 variants) across five classes: ‘*Benign*’ (35,659 variants, denoted by “2” label in calculations), ‘*Likely_benign*’ (21,931 variants, labelled as “1”), ‘*Uncertain_significance*’ (18,716 variants, labelled as “0”), ‘*Likely_pathogenic*’ (21,510 variants, labelled as “−1”), and ‘*Pathogenic*’ (35,698 variants, labelled as “−2”). Note that to improve the imbalance between the classes the original ClinVar categories *Benign/Likely_benign* and *Likely_benign* were merged into the ‘*Likely_benign*’ class, and similarly, ClinVar’s *Pathogenic/Likely_pathogenic* and *Likely_pathogenic* were merged into the ‘*Likely_pathogenic*’ class; the ‘*Uncertain_significance*’ class was down-sampled seven times randomly.

After training of the models of the ‘training’ dataset, cVEP classes were extended to the ‘prediction’ dataset, comprised of all remaining nsSNVs contained in the dbNSFP4.0, i.e., 84,013,490 human nsSNVs in the human coding genome split by the chromosome regions. The resulting output database is provided (link below). In addition, the results illustrate the dependence of the cVEP classifications on the indispensability, and differences in individual genes of two pathogenic types.

## 3. Results

### 3.1. Visualization of Variant Class Discrimination in 1- and 2-Dimensional Spaces

For the purpose of illustrating the usefulness of combining different scores, we show how the variants belonging to different pathogenicity classes can be progressively better discriminated, going from 1- to 2-dimensional space using one or two selected features, respectively. Subsequently, we extend the classification to the nD space (41 features).

#### 3.1.1. One-Dimensional Discrimination of ClinVar Variants by Using AAF, Indispensability, and VEST4 Annotations

**Figure 1:**
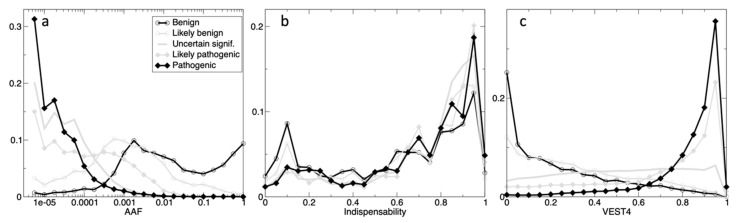
The histograms of the ClinVar’s classes in the dimensions: AAF (**a**), indispensability (**b**) and VEST4 (**c**). The counts are normalized to the total count (in five classes) in 20 bins (range 0-1, step 0.05) for the scores, and 24 bins for AAF (step 0.25 on the log_10_ scale). For AAF, the *x*-axis is on the log_10_-scale, e.g., 10^−2^ means that a variant can be found in 1 every 100 individuals. The five classes are: ‘*Benign*’ (thick open black circles), ‘*Likely_benign*’ (thin open grey circles), ‘*Uncertain_significance*’ (thick grey), ‘*Likely_pathogenic*’ (thin grey closed diamonds), and ‘*Pathogenic*’ (thick closed black diamonds).

Figure 1 displays three histograms for the five classes of gnomAD variants having ClinVar annotations, in dimensions: AAF, Indispensability, and VEST4, where the latter is the best performing functional annotation (see Section 3.2.4). For AAF (Figure 1a), there is a threshold clearly identifiable on the plot between the benign and the pathogenic classes at 0.0003, but the corresponding ‘*Likely*’ classes are more overlapped. The indispensability histograms on Figure 1b are very similar, but there are twice as many variants for pathogenic class in comparison to benign for low indispensability seen at 0.1, and the opposite is true at 0.95, meaning that there are twice as many benign variants for the mutation-tolerant genes (i.e., low indispensability), and twice as many pathogenic variants for essential genes (i.e., high indispensability). There is a very clear separation for VEST4 on Figure 1c, where pathogenic and benign (and the ‘*Likely*’ classes) can be distinguished by a threshold at 0.64 VEST4 score. Here, there is no differentiation for the ‘*Uncertain_significance*’ class.

#### 3.1.2. Discrimination of Variants by Pathogenicity Using the Population Allele Frequencies

**Figure 2:**
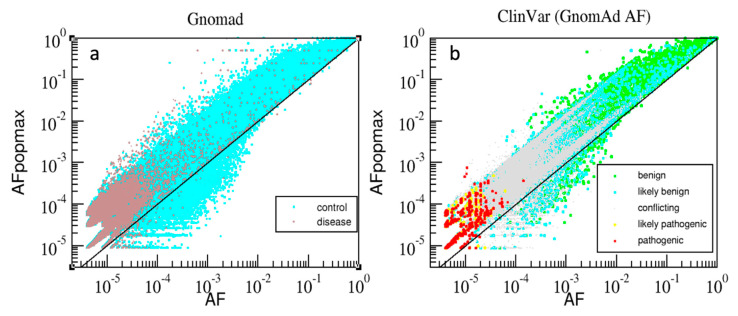
The gnomAD variants (**a**) are plotted in two groups–‘control’ (cyan) and ‘disease’ (brown). The variants with available ClinVar, AAF, AAF_popmax_ (**b**) are plotted by colored dots in five groups: ‘*Benign*’ (green), ‘*Likely_benign*’ (cyan), ‘*Conflicting*’ (grey), ‘*Likely_pathogenic*’ (yellow) and ‘*Pathogenic*’ (red); the dimensions (log_10_-scale): *x*-axis-AAF, and *y*-axis-AAF_popmax_.

AAF describes frequencies of nsSNPs at a gene locus. A population maximal AAF frequency (AAFpopmax [4]), is calculated as the maximum of AAF values across sub-populations. Figure 2 compares the distributions of variants in the dimensions AAF vs. AAF_popmax_ for the gnomAD and the ClinVar datasets. A variant is plotted above the diagonal when AAF_popmax_ > AAF, signifying that the variant has higher prevalence in some sub-population in comparison to the entire population. The ‘control’ variants (cyan colored dots) span the entire range of AAF above the diagonal; rare variants are found both above and below the diagonal to a similar extent. Conversely, ‘disease’ variants are mostly very rare (AAF range 10^−4^–10^−6^) and are situated above the diagonal. Similar to the ‘control’ group, the ClinVar annotations in the ‘*Benign*’ and ‘*Likely_benign*’ groups span the entire range of frequencies, while similarly to the ‘disease’ group, the ‘*Likely_pathogenic*’ and ‘*Pathogenic*’ groups are also clustered in the very rare range and above the diagonal. The ‘*Conflicting*’ group (ClinVar term ‘*Conflicting_pathogenicity_interpretations*’) has mostly intermediate frequency values. It is noteworthy that majority of both the ‘Disease’ (brown dots, Figure 2a) and ‘Pathogenic’ variants (red circles, Figure 2b) are above the diagonal. Consequently, the measure of difference (AAF_popmax_ − AAF > 0) is potentially useful (in combination with AAF < 10^−4^) as an additional measure to distinguish very rare pathogenic variants from very rare benign variants. This highlights the need for combining multiple scores by a machine learning approach that can automatically leverage such information.

**Figure 3:**
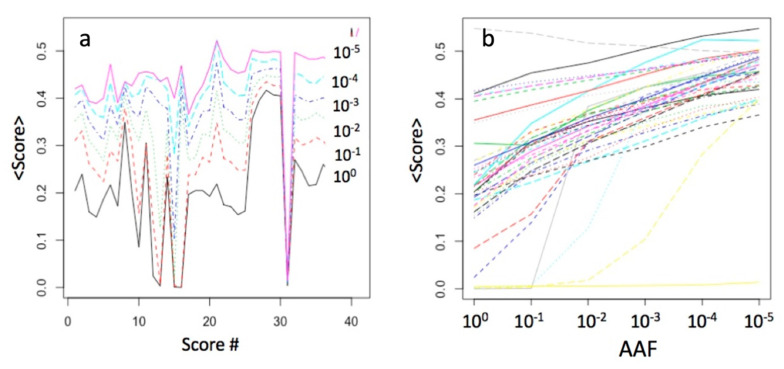
Comparison of the means of the 40 scores in the AAF ranges (log_10_-scale) for ca 5 million gnomAD variants: (**a**) the score means as a function of their sequential numbers; sorting corresponds to the sequential order in the dbNSFP, the same as in Table 1; (**b**) the same data transposed.

Each functional prediction tool had a converted rank-score, with the values ranging from 0 (benign) to 1 (pathogenic). Figure 3 shows the means of the 40 scores for the gnomAD dataset, computed for the six AAF ranges. As the ranges regress from common to very rare from bottom to top (Figure 3a), the score means increase, for example from around 0.2 for the range [1.0–0.1] to 0.5 for the range [10^−5^–10^−6^]. There is a close to linear relationship between the score mean values and the AAF value for most scores, while some had a step-function dependence (Figure 3b).

#### 3.1.3. Discrimination of Variants Using the Pathogenicity Scores

**Figure 4:**
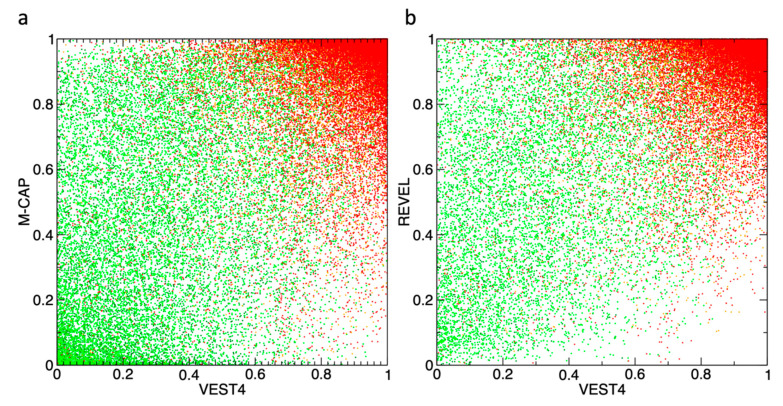
The variants with available ClinVar labels are plotted in two dimensions: *x*-axis–VEST4 scores, and *y*-axis–M-CAP (**a**) or REVEL (**b**) scores. The ClinVar classes: ‘*Benign*’ (dark green), ‘*Likely_benign*’ (green), ‘*Likely_pathogenic*’ (orange), and ‘*Pathogenic*’ (red).

Figure 4 illustrates the distribution of the variants having ClinVar annotations in two sets of the pathogenicity dimensions. The pathogenic variants (red, orange) are clustered in the top-right corner on both plots, while benign variants (dark-green, green) have a considerably greater spread across both panels, with noticeably better clustering in the bottom-left corner for the M-CAP vs. VEST4 2D (Figure 4a), in comparison to the REVEL vs. VEST4 (Figure 4b). Clearly, the 2D variant discrimination is more resolved compared to 1D, but there is still a large amount of overlap.

### 3.2. Training and Validation Using ClinVar Annotations

#### 3.2.1. Ranking of Predictive Capacity of Individual Pathogenicity Features

To obtain a ranking of the features using correlations between the score values and the target class values, we performed training with only “GLM” base-learner and only one feature, i.e., each of the scores individually. The rankings sorted by McFadden’s pseudo R-squared are given in Table 2. The most highly correlated score was ‘×10′ (VEST4) with R^2^ = 0.82, closely followed by ‘×20′, ‘×24′. The other R^2^ values were in the range of 0.75–0.81. It is interesting to note that the cross-species conservation scores tend to have lower R^2^ values in comparison to the functional scores, as can be inferred from their clustering in the second half of the table.

#### 3.2.2. Histogram of the Features by the ClinVar Classifications

**Figure 5:**
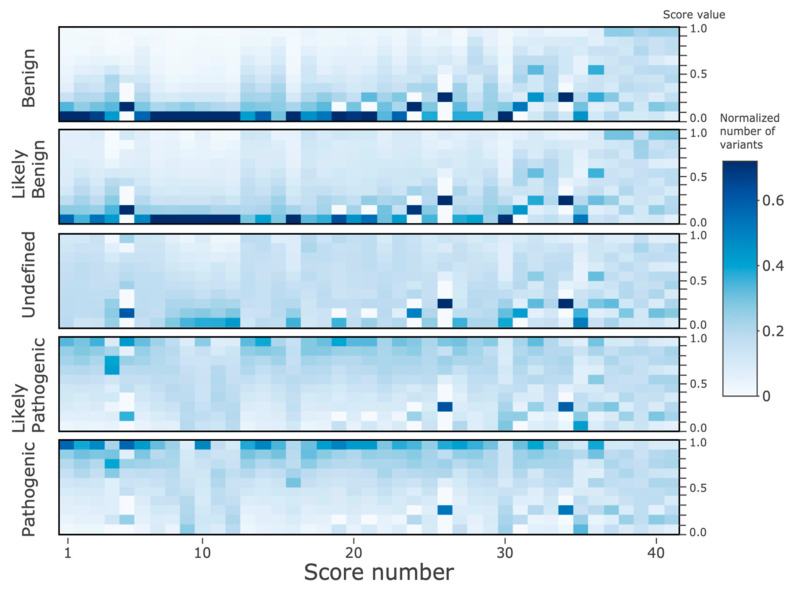
2D histogram of the score values of the ClinVar dataset. The *x*-axis displays the 41 features, sorted from left-to-right according to decreasing r^2^ as given in Table 2. The *y*-axis on the left side denotes the five ClinVar classes, and the *Y*-axis on the right corresponds to the histogram bins for the continuous values of each of 41 features (on the *x*-axis, 10 equally 0.1-spaced bins between 0 and 1). The colors from white (0) to blue (0.7) correspond to histogram counts (normalized to 1) in each of the bins.

Figure 5 shows 2D histograms of the 41 score values, divided into five ClinVar target classes (see *Methods*). To aid interpretation, the scores were sorted descending by their R^2^ along the *x*-axis (same as in Table 2). It is apparent that the colors in the left half of the plot are considerably more correlated both mutually and to the predicted class, in comparison to the right half, where the histogram patterns are much more random and less correlated. In particular, the ‘*Benign*’ and ‘*Likely_benign*’ classes in the left half have high histogram counts towards the bottom of the panels. In contrast, the ‘*Likely_pathogenic*’ and ‘*Pathogenic*’ classes have high variant counts towards the top of the panels. The histogram pattern in the ‘*Uncertain_significance*’ class in the left half resembles more the benign classes. Overall, the histogram patterns are highly correlated with the predicted class in the left half, while in the right half of the panels, the patterns are somewhat similarly poorly correlated.

#### 3.2.3. Training and Cross-Validation of the Ensemble Model

The method uses ‘training’ dataset as described in the Methods section. The performance measures of five-fold cross-validation (where ‘training’ dataset is was split into two parts: 80% training, 20% testing, with data points selected randomly using the ‘modulo’ algorithm from the *h2o* package) for the three base-learners (“GLM”, “GBM”, “DRF”) and the Super-learner (“Ensemble”) summarized in Table 3a. The *GLM* has the worst performance, *GBM* intermediate and *DRF* the highest among the base-learners. The accuracy is significantly better for *DRF* and *Ensemble* compared to the first two base-learners. The cross-validation runs (Table S3) point to the robustness and of the prediction of cVEP classes.

In particular, for *Ensemble*: *mean-per-class-error (mpce*) is 0.084. The number of misclassifications in five classes is 7.4% (according to ‘*Confusion Matrix*’ Table S3), with corresponding *“HR1”* of 92.5% for predicting the correct class. *“HR2”* is predicting either the correct or the nearest class is 98.8%. Since ‘next’ is along the class sequence (“−2” ‘*Pathogenic*’, “−1” ‘*Likely_pathogenic*’, “0” ‘*Uncertain_significance*’, “1” ‘*Likely_benign*’ and “2” ‘*Benign*’), misclassification into next-class is acceptable, while three- or more class misclassification is not (HR 3 to 5). The errors can be also attributable to ambiguity in the classes themselves, e.g., mixing between *likely* and *pure* classes. As can be seen from Table S3, most of the misclassifications occur between ‘*Pathogenic*’ and ‘*Likely_pathogenic*’, or between ‘*Likely_benign*’ and ‘*Benign*’. Besides, the inaccuracies are due to the errors in the input data and due to missing data for some scores (in the gnomAD database, the numbers of scores available for each variant range between minimum of 23 and maximum of 40).

Table 3b shows the accuracies for real-valued pathogenicity prediction. Here, the training/testing dataset was the same, except the predictive column was treated as real-values, i.e., −2, −1, 0, 1, 2 (instead of treating these as symbolic classes “−2”, “−1”,”0”,”1”,2”, as in the above). The training parameters were the same, except for the distribution type.

#### 3.2.4. Ranking of Features by Importance

**Figure 6:**
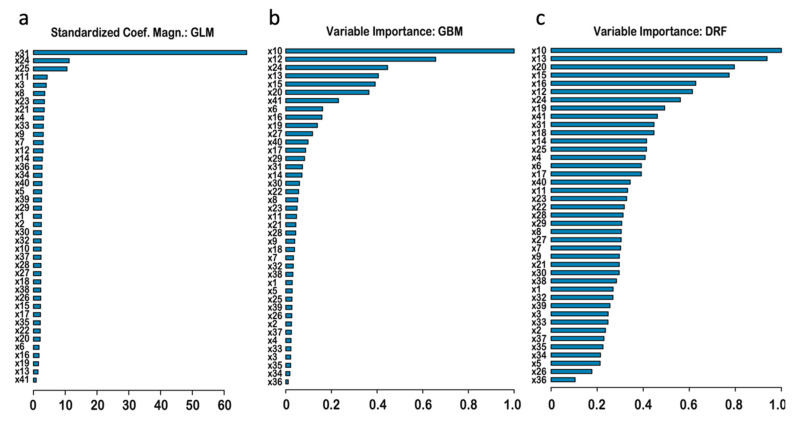
Ranking of the features by importance in the three base-learner models: GLM (**a**), GBM (**b**), and DRF (**c**), with importance decreasing from top to bottom.

Ranking by importance in the three base-models (Figure 6) showed which features gave the most important contributions. Since all of the individual scores correlations with target were high in the range 0.75–0.82 (Table 2), it is not surprising that the rankings show a high level of redundancy among the features, exhibited by only a fraction of scores having high importance. It is important that the base-models had different most contributing features, signifying good diversity among the base learners, needed for effectiveness of the super-learning algorithm. For the GLM model (Figure 6a), the most contributing feature by magnitude is *mean* (×31), followed by much smaller contributions from *Eigen-raw* (×24) and *Eigen-PC* (×25). For GBM (Figure 6b) the top-5 most important are: *VEST4*, *Meta-LR*, *Eigen-raw*, *CADD*, *MutPred* (×10, ×12, ×24, ×13, ×15). For DRF (Figure 6c), these are: *VEST4*, *CADD*, *M-CAP*, *MutPred*, *MVP* (×10, ×13, ×20, ×15, ×16).

The rankings provide three differing selections of the best scores for variant filtering. The GBM algorithm sequentially selects features, with the next most-contributing feature required to be orthogonal to the preceding features. Thus, the GBM list of top-ranking features has much lesser redundancy, and importance decreases quicker, compared to the DRF, where selection of the most-contributing features is done without such orthogonality requirement. It is interesting that the GLM top-ranks the ‘*Mean*’ feature, which is computed as an average of the features available for each variant. The mean score should indeed have theoretically lower by factor of sqrt (39) error level compared to each individual score feature used alone, due to averaging of noise, which led to its high rank in the linear regression model. However, this mean score is less important for GMB and DRF base-approaches, where ×31 was ranked 15th and 10th, respectively. In contrast to GLM ranking, the random forest methods have the ability to take into account all features giving unique significant contribution, especially so by DRF.

Gene indispensability (×41) is not directly correlated with pathogenicity as seen by its last 41th rank for GLM; nonetheless, its ranks for GBM and DRF are high: 7th and 9th, which signifies that the RF methods can make use of the important contribution of the feature. Here (as in Table 2), the majority of the cross-species conservation scores rank generally lower and cluster toward the tails of the graphs, with the exceptions of the *bStatistic* (×40), which had ranks in the middle range at 17th, 12th, and 17th positions in the three base-learners. The most contributing top-5 features listed above are proposed for use in variant filtering.

### 3.3. Prediction of Consensus Pathogenicity

#### 3.3.1. cVEP Values for all nsSNVs by Chromosomes

Table S4 shows very similar proportions of predicted cVEP classes for all nsSNVs grouped by the human chromosomes 1–22, X, Y, and M: around 34–41% and 11–22% in the ‘*Likely_benign*’ and ‘*Benign*’ classes, around 16–18% and 4–5% in the ‘*Pathogenic*’ and ‘*Likely_pathogenic*’ classes, respectively, and with a quarter in the ‘*Uncertain_significance*’ class. The situation is different for the Y and M chromosomes: Y has fewer pathogenic and more benign variants. It is especially notable that M has nearly no variants in the ‘*Likely*’ and ‘*Uncertain*’ classes, with all variants either ‘*Pathogenic*’ (40%) or ‘*Benign*’ (55%). It is worth noting that the ‘control’ dataset had higher proportions in the benign classes and by half less variants in the pathogenic classes, in comparison to all variants’ averages.

#### 3.3.2. Variation in cVEP by the Gene Indispensability

To assess differences in predicted variant pathogenicities as a function of the gene indispensability score *I*, the variants of the entire gnomAD dataset were split into three ranges (Table 4). The mutation-tolerant group (I < 0.3) has a considerably higher proportion of predicted benign or likely benign variants combined of around 80%. In contrast, the essential group (I > 0.95) has a much smaller proportion (8.5%) of predicted benign variants, with most classified as uncertain or likely benign. Sixty-six percent of the intermediate group’s variants were predicted to be as benign or likely benign.

#### 3.3.3. Application of cVEP to Pathogenic Genes

The proportion of variants predicted to be pathogenic in a selection of known pathogenic genes (Table 5) was as expected much higher than on average. The highest proportion of pathogenic or likely pathogenic variants for cardiac disorder genes was found for the MYH7 (61%) and FBN1 (52%) genes. The proportion of uncertain significance class is relatively large in this group, with the highest of 74% for DSP and 69% for PKP2. For recessive genes, the pathogenic proportion is highest for HBB (70%) and GJB2 (62%).

## 4. Discussion

The main advantage of the proposed approach is of a practical computational nature. We do not suggest replacing the plethora of existing scores in favor of one new score. On the contrary, we suggested above the use of a combination of several top-ranking scores, which were shown to have the highest predictive capacity. Nonetheless, the advantage of using a consensus score becomes very clear when attempting to sort a list of sequenced WES variants by several pathogenicity scores. When sorting separately by several scores in a sequence of steps, and applying a realistic threshold (e.g., 0.5) for filtering on each step, usually all variants become completely filtered out after several steps. Here we overcome this challenge and provide a means of ‘sorting’ any subset of variants (and without using any thresholds), because the consensus score has incorporated contributions from all used scores. To give quantitative references: in the consensus approach, since the correlations between the target annotations and each individual score are in the 75–82% range (Table 2), and since the cVEP predictions have 98% match with the target, the resulting annotations are in line with the input scores to the nearly same degree as the targets (i.e., 73–80% correlation). The method minimized the prediction errors of each input feature, thus producing for the consensus several times smaller error values, i.e., smaller false-positive (FP) and false-negative (FN) rates. The quantitative comparison of the recent methods given here [15] allows directly comparing the FP/FN-rates of other methods with the cVEP rates obtained here, since many of them (with the exception of REVEL, which is trained on a dataset containing many new rare variants [9]) are also trained on the ClinVar dataset. This study [15] cited FP rates in the 10–40% range for several very popular scores (VEST3, metaLR, metaSVM, M-CAP, fathm-MKL, Eigen, GenoCanyon, REVEL); in contrast, 2% FP error was observed for the cVEP 2-class classification, which is much smaller. The improvement in error is achieved by using the super-learning two-level method with base-learners having different depths, and by taking into account the importance and redundancy of each score’s contribution to the target prediction.

Another novelty here is that the method finds a consensus of all continuous value scores in the form of a categorical value, which is compatible with the ACMG/AMP standards and guidelines for use in clinical practice. This categorical classification has the advantage of allowing its direct use in WES applications for performing one-score ranking of variants without any thresholds. Such an approach was proposed long ago by researchers in this field, but the present paper makes a meaningful step towards implementing it for the first time. This categorization is a definite improvement, allowing for more than just binary (pathogenic, benign) classifications and including ‘likely’ and ‘uncertain’ classes, thereby addressing biological complexities somewhat better.

A novel gene indispensability score was included in the predictors for several important reasons. On the one hand, there are a considerable number of potentially deleterious variants (i.e., those having high values for their continuous pathogenicity scores) located in mutation-tolerant genes. Such variants that also have high population allele frequencies could be assumed neutral based on that measure alone, and, consequently, could be associated with no negative effects in cells; as a result, they could be grouped into the ‘*Benign*’ class in the ClinVar database. On the other hand, moderately deleterious variants (i.e., those having medium-level pathogenicity scores) in essential genes can be potentially associated with very adverse effects in cells, and thus included in the ‘*Pathogenic*’ ClinVar class. A relatively large number of such cases could be a major source of error when training an algorithm based on pathogenicity scores alone. However, here such effects are partially accounted for by the indispensability score, and it is not surprising that this score contributed significantly to the improvement in accuracy; when ranking features by importance, that score (x41) was listed in the top ten. The addition of the indispensability has reduced the total amount of classification errors more than five-fold (Table S5): from an initial ca. 10% (without *Indispensability*) to the current ca. 2%. Similarly, this work obtained feature rankings by importance for the first time, which can be used as a guide when selecting scores for variant filtering.

To list limitations of the approach, first of all, it would clearly be preferable to use annotations based on actual clinical or experimental evidence from in vitro or in vivo test results to determine variant effects instead of any prediction method. However, such data are currently unavailable for more than 99% of all potential nsSNVs. This situation is very likely to improve in the future; for example, it might become possible to obtain direct measures of pathogenicity using high-throughput in vitro cell screening assays of synthetic mutations, e.g., using the CRISPR approach [29], leading to a rapid increase in the percentage of evidence annotations. However, there are some types of variants, such as null mutations, that appear to be too pathogenic to exist in a living cell or whose effects might remain below the screen’s detectability level.

Secondly, it is important to note that as with other similar tools, this method cannot replace methods that are currently mainstream for distinguishing pathogenic somatic variants from germline ones, e.g., using sequence data from family trios, or from direct evidence on variant-disease association, or information on genomic structure.

Thirdly, it is important to note the limitation of using ClinVar annotations for training. In GWAS studies, when a causal and common connection between clinical phenotype and genotype is derived initially from a small cohort, the results frequently become invalid or are contradicted when more data is added from new studies, leading to these contradictory annotations subsequently becoming reclassified in ClinVar [Song 2016]. The current state of the continuous classification process can be seen in the (re)distribution of the classes of ClinVar annotations, including undefined or contradictory interpretations. Nonetheless, since the ACMG/AMP expanded guidelines require certainty of better than 90% from experimental clinical/biological evidence (the remaining <10% may be (re)classified as contradictory or uncertain), the large majority of the resulting database is not contradictory. In fact, it is well-established that the pathogenic/benign/likely status annotations derived from evidence-based data submitted to ClinVar are very useful for variant filtering and ranking. In this work, we ‘propagate’ using machine-learning techniques, the knowledge from known variant impacts into the unknown, thus covering all variants with predicted annotations.

Fourthly, annotation with pathogenicity is just one biologically-relevant dimension; others include the connections and associations of a variant to diverse phenotypes and diseases, and annotations like population allele frequency. Interpretation of variant impact is afflicted by multiple levels of complexity due to diverse direct and indirect influences on the effects of a mutation, to the interconnectedness of cell processes in an organism, and to the accumulation of multiple effects during the life of an organism or over the long course of evolution. However, such complex topics are mostly beyond the scope of this work. Nonetheless, we partially explored here the evolutionary connection between three characteristics of a gene variant: allele frequency, mammalian family conservation, and pathogenicity effects. It is undeniable that pathogenic variants are less conserved in families and less frequent in genetic sequences, while beneficial and benign mutations are more prevalent in populations.

Finally, new evidence and bioinformatic databases continuously appear that address the question of variant impact from new and different angles; perhaps, the future will bring more direct and more effective approaches. In this study, we focused only on the scores that are explicitly termed ‘pathogenicity prediction’ and on non-synonymous exome SNPs. Adding gain/loss-of-function (GOF/LOF) annotation might improve computational interpretation of variant effects. Similar to nsSNV mutations, GOF/LOF for a variant is not directly causative of pathogenicity, as these can lead to all types of impacts—making effects by such mutations genes more or less pathogenic, or neutral. Currently, GOF/LOF annotation remains challenging or incomplete; it remains to be seen whether it will be possible to add these scores to the combination, and whether doing so will provide improvement of accuracy, or if such scores should only be used separately. Although our approach is extensible to adding short slice-site exome mutations, regulatory and other variants in non-coding areas, but such future work would require a different set of input scores, and, possibly, a different approach, e.g., doing it in groups by diseases or traits. One such example of non-coding variant annotation methods, DIVAN [30] is an ensemble learning framework with feature selection. It is able to identify non-coding disease-specific risk variants using thousands of epigenomic annotations, such as histone marks. It evaluates and annotates variants with respect to 45 different diseases/traits.

Although the results of this work are immediately useful, we suggest treating this attempt to classify all variants as exploratory, and to use the predictions with a degree of caution similar as for other predictive scores. The cVEP labels should be considered putative and treated as predictions only, which should not to be confused with the annotations based on actual evidence from clinical and biological data, such as in ClinVar database. In contrast to the pathogenicity scores, produced by a number of other predictive tools in the form of a continuous value ranging from 0 to 1 (e.g., 0.65), we produced the consensus predictions as five multinomial classes corresponding to the respective ClinVar labels. This is done to facilitate for the reader interpretation of the results in this work, but we did not intend to claim that we produced evidence level annotations. All told, with the advantages of the approach summarized below, the predictions might be especially useful for the large number of ultra-rare and singleton germline or rare somatic variants, which have no AAF or biological/clinical evidence due to their rarity.

## 5. Conclusions

In this work, we developed a new approach for addressing an important and persistent challenge: the annotation of coding genome variants with predicted mutation impact effects, but for which biological evidence is lacking. To achieve this, we implemented a novel super-learning approach based on a set of base-learners with different abilities in the hierarchical classification of variants, thereby combining information from a large set of variant annotations. This combination approach is characterized by improved accuracy of variant-effect predictions. The addition of the gene indispensability score, which is based on domain knowledge of several cellular networks, along with accounting for gene differences in essentiality and mutation-tolerance, contributed significantly to improving the predictions. We showed that all of the functional, conservation, and impact scores used in this study correlate with the available pathogenicity evidence, and also anti-correlate with human population allele frequencies. These findings suggest that using these features in a combination approach is an appropriate method of predicting variant pathogenicity. Assessment of the performance of the method using five-fold cross-validation on the ClinVar dataset confirmed the reliability and robustness of its predictions. Finally, the resulting putative annotations for the exome SNVs are aimed at helping to distinguish between pathogenic, uncertain-significance, and benign variants in biological and clinical WES applications.

## Figures and Tables

**Table 1:** List of the scores used *.

x	Score Name
1	SIFT_converted
2	SIFT4G_converted
3	Polyphen2_HDIV
4	Polyphen2_HVAR
5	LRT_converted_
6	MutationTaster_converted
7	MutationAssessor
8	FATHMM_converted
9	PROVEAN_converted
10	VEST4
11	MetaSVM
12	MetaLR
13	M-CAP
14	REVEL
15	MutPred
16	MVP
17	MPC
18	PrimateAI
19	DEOGEN2
20	CADD_raw
21	DANN_
22	fathmm-MKL_coding
23	fathmm-XF_coding_
24	Eigen-raw_coding
25	Eigen-PC-raw_coding
26	GenoCanyon_score
27	integrated_fitCons
28	GM12878_fitCons
29	H1-hESC_fitCons
30	HUVEC_fitCons
31	Mean
32	GERP++_RS
33	phyloP100way_vertebrate
34	phyloP30way_mammalian
35	phyloP17way_primate
36	phastCons100way_vertebrate
37	phastCons30way_mammalian
38	phastCons17way_primate
39	SiPhy_29way_logOdds
40	bStatistic
41	Gene_indispensability

* The scores numbers correspond to their sequential order in the dbNSFP4.0: 1–30 functional scores, 31—mean score, 32–40 conservation scores, 41 indispensability; the rank (converted) versions are used (the sources are provided in Supplementary Materials).

**Table 2:** Ranking of features sorted descending by pseudo-*R*^2^.

×10 0.818 VEST4	×22 0.790 fathmmMKL	* ×32 0.775 phyloP100vertebrate
×20 0.811 CADD	×11 0.789 MetaSVM	×8 0.771 fathmm
×24 0.811 Eigen	×17 0.787 MPC	* ×37 0.770 phastCons30mammal
×31 0.808 Mean	×9 0.787 Provean	* ×34 0.768 phyloP30mammalian
×6 0.806 MutationTaster	×3 0.786 Polypen2HDIV	* ×38 0.767 phastCons17primate
×25 0.805 Eigen-PC	×7 0.786 MutationAssessor	×26 0.765 Geno Canyon
×15 0.799 MutPred	×1 0.785 SIFT	×41 0.763 Gene_indispensability
×14 0.798 REVEL	* ×33 0.784 phyloP100vertebrate	* ×35 0.762 phyloP17wayprimate
×13 0.798 M-CAP	×2 0.781 SIFT4G	×27 0.751 integrated_fitCons
×12 0.797 MetaLR	×5 0.781 LRT	×29 0.750 H1-hESC_fitCons
×19 0.793 Deogen2	* ×39 0.781 SiPhy29waylogOdds	* ×40 0.750 bStatistic
×16 0.793 MVP	* ×36 0.780 phastCons100vertebr	×30 0.750 HUVEC_fitCons
×18 0.792 PrimateAI	×21 0.779 DANN	×28 0.750 GM12878_fitCons
×4 0.791 Polyphen2HVAR	×23 0.779 fathmmXF	

* pure conservation score.

**Table 3:** (**a**) Summary of test-set metrics for cVEP class prediction. (**b**) Summary of test-set metrics for continuous-value prediction.

(**a**)				
**Metric**	**GLM**	**GBM**	**DRF**	**Ensemble**
mse	0.39	0.21	0.12	0.10
rmse	0.62	0.46	0.34	0.32
logloss	1.06	0.65	0.39	0.36
mpce	0.53	0.29	0.074	0.084
HR1	0.55	0.74	0.939	0.925
HR2	0.83	0.93	0.997	0.988
(**b**)				
**Metric**	**GLM**	**GBM**	**DRF**	**Ensemble**
mse	0.79	0.50	0.55	0.24
rmse	0.89	0.71	0.74	0.49
mae	0.62	0.52	0.55	0.36
mrd	0.79	0.50	0.55	0.23
R_c_^2^	0.82	0.89	0.957	0.952

Mse, mean standard error; rmse, root mean square error; logloss, logarithmic loss; mpce, Mean Per-Class Error; HR1, within class; HR2, within 2 classes; mae, mean average error; mrd, mean residual deviation; R_c_, Pearson’s correlation coefficient.

**Table 4:** Proportions of predicted pathogenicity classes for three gene indispensability ranges.

Gene Indispensability Ranges	Pathogenic	Likely_Pathogenic	Uncertain	Likely_Benign	Benign
mutation-tolerant genes 0.0 < I < 0.3	0.105	0.010	0.091	0.489	0.305
intermediate I genes 0.3 < I < 0.7	0.122	0.017	0.197	0.499	0.165
essential genes 0.95 < I < 1.0	0.104	0.038	0.439	0.335	0.085

**Table 5:** Proportions of pathogenicity classes for the selected pathogenic genes.

Genes	Pathogenic	Likely_Pathogenic	Uncertain	Likely_Benign	Benign
Cardiac disorder genes					
fbn1	0.156	0.362	0.430	0.051	0.001
myh7	0.103	0.504	0.374	0.020	0.000
tnni3	0.187	0.203	0.484	0.085	0.042
tnnt2	0.152	0.259	0.532	0.041	0.017
mybpc3	0.166	0.098	0.566	0.163	0.007
dsg2	0.110	0.021	0.580	0.274	0.015
pkp2	0.127	0.048	0.686	0.133	0.006
dsp	0.099	0.036	0.743	0.118	0.004
dsc2	0.160	0.023	0.452	0.339	0.026
Recessive genes					
hbb	0.694	0.013	0.163	0.095	0.035
gjb2	0.333	0.284	0.349	0.031	0.004
cftr	0.320	0.160	0.473	0.042	0.005
mefv	0.124	0.009	0.227	0.563	0.077

## Supplementary Materials

The following are available online at https://www.mdpi.com/2073-4425/11/9/1076/s1, Table S1 with the list of the impact effect scores used for input in the machine learning procedure; Table S2 with the individual parameters of the base- and meta-learners; Table S3a–e with the outputs of the model fits containing cross-validations metrics for the base- and ensemble-learners; Table S4 with proportions of cVEP classes of the nsSNVs grouped by the chromosomes; Table S5 with improvement in predictions accuracy by including indispensability. Availability: the predicted consensus VEP (cVEP) class scores for the 84,013,118 human exome nsSNVs are available here: https://cvep.imbi.uni-freiburg.de/cvep.tgz, in the form of 1.2Gb tar-gzip archive, in BED (Browser Extensible Data) format split by the chromosome regions; the archive can be searched using provided Java program by input of hg19, hg38 (1-based coordinates) or gene, chromosome; it is compatible with any of the variant annotation tools that can use the dbNSFP database Version 4.0.
