# Supplementary material for: Annotation of Human Exome Gene Variants with Consensus Pathogenicity
Source: Genes (Basel). 2020 Sep 14;11(9):1076. doi: 10.3390/genes11091076 (PMC7563776; doi:10.3390/genes11091076)
Supplement: Supplementary file 1 [file genes-11-01076-s001.pdf]

Supplemental information

Table S1. List of the impact effect scores.

dbnsfp-ID	*Score name*	*Source*
38	SIFT_converted_rankscore	SIFT ensembl 66, released Jan, 2015 http://provean.jcvi.org/index.php
41	SIFT4G_converted_rankscore	SIFT4G 2.4, released Nov. 1, 2016 http://sift.bii.a-star.edu.sg/sift4g/public//Homo_sapiens/
44	Polyphen2_HDIV_rankscore	Polyphen-2 v2.2.2, released Feb, 2012 http://genetics.bwh.harvard.edu/pph2/
47	Polyphen2_HVAR_rankscore	Polyphen-2 v2.2.2, released Feb, 2012 http://genetics.bwh.harvard.edu/pph2/
50	LRT_converted_rankscore	LRT, released November, 2009 http://www.genetics.wustl.edu/jflab/lrt_query.html
54	MutationTaster_converted_rankscore	MutationTaster 2, data retrieved in 2015 http://www.mutationtaster.org/
59	MutationAssessor_rankscore	MutationAssessor release 3, http://mutationassessor.org/
62	FATHMM_converted_rankscore	FATHMM v2.3, http://fathmm.biocompute.org.uk
65	PROVEAN_converted_rankscore	PROVEAN 1.1 ensembl 66, released Jan, 2015 http://provean.jcvi.org/index.php
68	VEST4_rankscore	VEST v4.0, http://karchinlab.org/apps/appVest.html
70	MetaSVM_rankscore	MetaSVM and MetaLR, doi: 10.1093/hmg/ddu733
73	MetaLR_rankscore	MetaSVM and MetaLR, doi: 10.1093/hmg/ddu733
77	M-CAP_rankscore	M-CAP v1.3, http://bejerano.stanford.edu/MCAP/
80	REVEL_rankscore	REVEL, https://sites.google.com/site/revelgenomics/
82	MutPred_rankscore	MutPred v1.2, http://mutpred.mutdb.org/
87	MVP_rankscore	MVP 1.0, https://github.com/ShenLab/missense
89	MPC_rankscore	MPC release1, ftp://ftp.broadinstitute.org/pub/ExAC_release/release1/regional_missense_constraint/
91	PrimateAI_rankscore	PrimateAI, https://github.com/Illumina/PrimateAI
94	DEOGEN2_rankscore	deogen2, https://deogen2.mutaframe.com/
103	CADD_raw_rankscore	CADD v1.4, http://cadd.gs.washington.edu/
106	DANN_rankscore	DANN, https://cbcl.ics.uci.edu/public_data/DANN/
108	fathmm-MKL_coding_rankscore	fathmm-MKL, http://fathmm.biocompute.org.uk/fathmmMKL.htm
112	fathmm-XF_coding_rankscore	fathmm-XF, http://fathmm.biocompute.org.uk/fathmm-xf/
115	Eigen-raw_coding_rankscore	Eigen & Eigen PC v1.1, http://www.columbia.edu/~ii2135/eigen.html
118	Eigen-PC-raw_coding_rankscore	Eigen & Eigen PC v1.1, http://www.columbia.edu/~ii2135/eigen.html

121	GenoCanyon_score_rankscore	GenoCanyon v1.0.3, http://genocanyon.med.yale.edu/index.html
123	integrated_fitCons_rankscore	fitCons v1.01, http://compgen.bscb.cornell.edu/fitCons/
126	GM12878_fitCons_rankscore	fitCons v1.01, http://compgen.bscb.cornell.edu/fitCons/
129	H1-hESC_fitCons_rankscore	fitCons v1.01, http://compgen.bscb.cornell.edu/fitCons/
132	HUVEC_fitCons_rankscore	fitCons v1.01, http://compgen.bscb.cornell.edu/fitCons/
135	LINSIGHT_rankscore	LINSIGHT, http://compgen.cshl.edu/~yihuang/LINSIGHT/
138	GERP++_RS_rankscore	GERP++ http://mendel.stanford.edu/SidowLab/downloads/gerp/
140	phyloP100way_vertebrate_rankscore	phyloP100way_vertebrate (hg38) http://hgdownload.soe.ucsc.edu/goldenPath/hg38/phyloP100way/
142	phyloP30way_mammalian_rankscore	phyloP30way_mammalian (hg38) http://hgdownload.soe.ucsc.edu/goldenPath/hg38/phyloP30way/
144	phyloP17way_primate_rankscore	phyloP17way_primate (hg38) http://hgdownload.soe.ucsc.edu/goldenPath/hg38/phyloP17way/
146	phastCons100way_vertebrate_rankscore	phastCons100way_vertebrate (hg38) http://hgdownload.soe.ucsc.edu/goldenPath/hg38/phastCons100way/
148	phastCons30way_mammalian_rankscore	phastCons30way_mammalian (hg38) http://hgdownload.soe.ucsc.edu/goldenPath/hg38/phastCons30way/
150	phastCons17way_primate_rankscore	phastCons17way_primate (hg38) http://hgdownload.soe.ucsc.edu/goldenPath/hg38/phastCons17way/
153	SiPhy_29way_logOdds_rankscore	SiPhy https://www.broadinstitute.org/mammals-models/29-mammals-project-supplementary-info
155	bStatistic_rankscore	bStatistic http://cadd.gs.washington.edu/
227	gnomAD_exomes_AC	Alternative allele count in the whole gnomAD exome samples (125,748 samples)
261	gnomAD_exomes_POPMAX_AF	Maximum allele frequency across populations (excluding samples of Ashkenazi, Finnish, and indeterminate ancestry)
263	gnomAD_exomes_controls_AC	Alternative allele count in the controls subset of whole gnomAD exome samples (54,704 samples)
297	gnomAD_exomes_controls_POPMAX_AF	Maximum allele frequency across populations (excluding samples of Ashkenazi, Finnish, and indeterminate ancestry) in the controls subset
463	Gene_indispensability_score	A probability prediction of the gene being essential. From doi:10.1371/journal.pcbi.1002886

The table contains dbNSFP IDs, score names and sources of the functional/conservation scores from dbNSFP4.0 (Liu X, Wu C, Li C and Boerwinkle E. 2016. dbNSFP v3.0: A One-Stop Database of Functional Predictions and Annotations for Human Non-synonymous and Splice Site SNVs. *Human Mutation.* 37(3):235-241.).

Table S2. The individual parameters of the base learners

(a) GLM: *function = h2o.glm, distribution = "multinomial", compute_p_values=FALSE, link="family_default", lambda=2e-6, solver="AUTO", balance_classes = TRUE, max_after_balance_size = 1.5, nfolds=5, fold_assignment="Modulo", keep_cross_validation_predictions = TRUE.*

(b) GBM: *function = h2o.gbm, ntrees = 30, max_depth = 6, learn_rate = 0.9, min_rows = 64, distribution="multinomial", balance_classes = TRUE, max_after_balance_size = 1.5, nfolds = 5, fold_assignment = "Modulo", keep_cross_validation_predictions = TRUE,.*

(c) DRF: *function = h2o.randomForest, ntrees = 130, max_depth = 20, sample_rate = 0.9, mtries = -1, min_rows = 5, balance_classes = TRUE, max_after_balance_size = 1.5,nfolds = 5, fold_assignment = "Modulo", keep_cross_validation_predictions = TRUE.*

(d) Ensemble: *function = h2o.stackedEnsemble, base_models=list(drf, gbm, glm), metalearner_nfolds=5, metalearner_fold_assignment="Modulo", metalearner_algorithm = "GLM").*

All other parameters were set to default values.

Table S3a. Model fitting output for the base-learner "GLM"

Model Details:
H2OMultinomialModel: glm
GLM Model: summary
Family= multinomial link= multinomial regularization= Elastic Net (alpha = 0.5, lambda = 3.0E−6)

Cross-Validation Set Metrics:

MSE: 0.388
RMSE: 0.623
Logloss: 1.06
Mean Per-Class Error: 0.535
R^2: 0.842

Top-5 Hit RatioTable:

	k	hit_ratio
1	1	0.545718
2	2	0.825097
3	3	0.937018
4	4	0.976519
5	5	1.000000

Cross-Validation Metrics Summary:

	mean	sd	cv_1_valid	cv_2_valid	cv_3_valid	cv_4_valid	cv_5_valid
accuracy	0.54080003	0.0022095032	0.541804	0.5437157	0.5408064	0.54281193	0.53486186

err	0.4592	0.0022095032	0.45819598	0.45628425	0.45919356	0.45718804	0.46513814
err_count	12261.8	56.784504	12232.0	12238.0	12322.0	12142.0	12375.0
logloss	1.0697118	0.0029180122	1.0699316	1.0686098	1.068005	1.0647764	1.077236
max_per_class_error	0.84432226	0.004410384	0.8384401	0.84464926	0.83900857	0.84373564	0.8557778
mean_per_class_accuracy	0.45626974	0.0016800825	0.45730522	0.45903042	0.45642695	0.45671114	0.451875
mean_per_class_error	0.54373026	0.0016800825	0.5426948	0.54096955	0.543573	0.5432889	0.54812497
mse	0.3948089	9.211634E-4	0.39526433	0.39438918	0.39470974	0.39282942	0.39685184
null_deviance	83932.414	252.94965	83970.7	84308.92	84314.74	83405.945	83661.74
r2	0.8397035	5.888411E-4	0.8390949	0.83982146	0.83998936	0.84102666	0.83858526
residual_deviance	57128.45	209.03355	57125.79	57322.367	57317.688	56556.664	57319.727
rmse	0.6283373	7.32975E-4	0.6287005	0.62800413	0.6282593	0.626761	0.6299618

Test Set Metrics:

MSE: 0.388
RMSE: 0.623
Logloss: 1.06
Mean Per-Class Error: 0.535
R^2: 0.842

Confusion Matrix:

	−2	−1	0	1	2	Error	Rate
−2	29355	3566	1349	333	1095	0.1777 =	6,343 / 35,698
−1	15212	4326	1195	213	564	0.7989 =	17,184 / 21,510
0	5544	1150	5434	2572	4016	0.7097 =	13,282 / 18,716
1	2684	482	3016	3500	12249	0.8404 =	18,431 / 21,931
2	1868	258	1450	1837	30246	0.1518 =	5,413 / 35,659
Totals	54663	9782	12444	8455	48170	0.4543 =	60,653 / 133,514

Row labels: Actual class; Column labels: Predicted class

Table S3b. GLM – sorted by pseudo-R2-measure from training using individual features

"10"	0.818
"20"	0.811
"24"	0.811
"31"	0.808
" 6"	0.806
"25"	0.805
"15"	0.799
"14"	0.798
"13"	0.798
"12"	0.797
"19"	0.793
"16"	0.793

"18" 0.792
" 4" 0.791
"22" 0.790
"11" 0.789
"17" 0.787
" 9" 0.787
" 3" 0.786
" 7" 0.786
" 1" 0.785
"33" 0.784
" 2" 0.781
" 5" 0.781
"39" 0.781
"36" 0.780
"21" 0.779
"23" 0.779
"32" 0.775
" 8" 0.771
"37" 0.770
"34" 0.768
"38" 0.767
"26" 0.765
"41" 0.763
"35" 0.762
"27" 0.751
"29" 0.750
"40" 0.750
"30" 0.750
"28" 0.750

Table S3c. Model fitting output for the base-learner “GBM”

Model Details:
H2OMultinomialModel: gbm
Model Summary:
number_of_trees number_of_internal_trees model_size_in_bytes min_depth
1 30 150 116246 7
max_depth mean_depth min_leaves max_leaves mean_leaves
1 7 7.00000 22 97 57.08000

Training Set Metrics:

MSE: 0.2316003
RMSE: 0.4812487
Logloss: 0.6917729

Mean Per-Class Error: 0.2778084

Confusion Matrix: Row labels: Actual class; Column labels: Predicted class

	−2	−1	0	1	2	Error	Rate
−2	29419	3898	1217	754	410	0.1759 =	6,279 / 35,698
−1	11205	21386	1764	938	372	0.4004 =	14,279 / 35,665
0	3286	1861	24325	4355	1885	0.3189 =	11,387 / 35,712
1	1332	600	3617	22671	7454	0.3645 =	13,003 / 35,674
2	472	143	952	3053	31074	0.1294 =	4,620 / 35,694
Totals	45714	27888	31875	31771	41195	0.2778 =	49,568 / 178,443

Hit Ratio Table:

Top-5 Hit Ratios:

	k	hit_ratio
1	1	0.722219
2	2	0.921706
3	3	0.974283
4	4	0.991667
5	5	1.000000

Cross-Validation Set Metrics:

MSE: 0.3138585
RMSE: 0.5602308
Logloss: 0.9818054
Mean Per-Class Error: 0.4377324

Top-5 Hit Ratios:

	k	hit_ratio
1	1	0.606401
2	2	0.869939
3	3	0.955038
4	4	0.983740
5	5	1.000000

Cross-Validation Metrics Summary:

	mean	sd	cv_1_valid	cv_2_valid	cv_3_valid	cv_4_valid	cv_5_valid
accuracy	0.6063998	9.945707E-4	0.60585856	0.60840386	0.605575	0.6076135	0.60454804
err	0.3936002	9.945707E-4	0.39414144	0.39159614	0.39442497	0.39238647	0.395452
err_count	10510.2	37.0281	10522.0	10503.0	10584.0	10421.0	10521.0
logloss	0.9817988	0.004505569	0.9781501	0.9850682	0.9820777	0.97239083	0.9913073
max_per_class_error	0.6004519	0.0018044758	0.6049496	0.59750646	0.59886366	0.59971714	0.6012227
mean_per_class_accuracy	0.5622839	8.974609E-4	0.56135696	0.56460136	0.56224	0.5622871	0.5609339
mean_per_class_error	0.43771613	8.974609E-4	0.43864304	0.43539864	0.43776	0.43771288	0.43906608

mse	0.31385753	0.0010074427	0.31441486	0.31337523	0.3141749	0.3114901	0.31583253
r2	0.87257034	5.740594E-4	0.87200725	0.8727247	0.8726372	0.8739437	0.8715389
rmse	0.56022847	8.9952536E-4	0.5607271	0.5597993	0.5605131	0.558113	0.5619898

Test Set Metrics:

MSE: 0.2147934
RMSE: 0.4634581
Logloss: 0.6485677
Mean Per-Class Error: 0.2849858

Confusion Matrix: Row labels: Actual class; Column labels: Predicted class

	−2	−1	0	1	2	Error	Rate
−2	29419	3898	1217	754	410	0.1759 =	6,279 / 35,698
−1	7008	12592	1110	572	228	0.4146 =	8,918 / 21,510
0	1727	986	12668	2340	995	0.3231 =	6,048 / 18,716
1	838	374	2364	13558	4797	0.3818 =	8,373 / 21,931
2	472	142	951	3053	31041	0.1295 =	4,618 / 35,659
Totals	39464	17992	18310	20277	37471	0.2564 =	34,236 / 133,514

Hit Ratio Table:
Top-5 Hit Ratios:

	k	hit_ratio
1	1	0.743578
2	2	0.927064
3	3	0.974482
4	4	0.991566
5	5	1.000000

Table S3d Model fitting output for the base-learner "DRF"

Model Summary:

	number_of_trees	number_of_internal_trees	model_size_in_bytes	min_depth
1	100	500	68228728	21

	max_depth	mean_depth	min_leaves	max_leaves	mean_leaves
1	21	21.00000	8039	13077	10881.03400

Training Set Metrics:

MSE: 0.245335
RMSE: (0.4953131

Logloss: 0.7839877
Mean Per-Class Error: 0.2541096

Confusion Matrix: Row labels: Actual class; Column labels: Predicted class

	−2	−1	0	1	2	Error	Rate
−2	30059	3405	1080	552	601	0.1579 =	5,638 / 35,697
−1	12753	21442	706	389	374	0.3988 =	14,222 / 35,664
0	3403	533	28036	1441	2298	0.2149 =	7,675 / 35,711
1	1213	197	1490	22560	10213	0.3676 =	13,113 / 35,673
2	637	122	825	3103	31004	0.1313 =	4,687 / 35,691
Totals	48065	25699	32137	28045	44490	0.2541 =	45,335 / 178,436

Hit Ratio Table:
Top-5 Hit Ratios:

	k	hit_ratio
1	1	0.745931
2	2	0.947455
3	3	0.981641
4	4	0.992171
5	5	1.000000

** 5-fold cross-validation on training data **

Cross-Validation Set Metrics:

MSE: 0.3121071
RMSE: 0.5586655
Logloss: 0.8790636
Mean Per-Class Error: 0.4533674

Top-5 Hit Ratios:

	k	hit_ratio
1	1	0.618946
2	2	0.883757
3	3	0.962723
4	4	0.986324
5	5	1.000000

Cross-Validation Metrics Summary:

	mean	sd	cv_1_valid	cv_2_valid	cv_3_valid	cv_4_valid	cv_5_valid
accuracy	0.61894226	0.0017818131	0.6192688	0.62260914	0.6179474	0.6199638	0.614922
err	0.38105777	0.0017818131	0.3807312	0.37739086	0.38205263	0.38003615	0.38507798
err_count	10175.2	45.257927	10164.0	10122.0	10252.0	10093.0	10245.0
logloss	0.87906915	0.002845447	0.8802153	0.87902075	0.8746687	0.87548447	0.8859566
max_per_class_error	0.7423255	0.004905025	0.7477085	0.7426488	0.72886366	0.7458746	0.74653184
mean_per_class_accuracy	0.54661924	0.0016683937	0.5476766	0.55073774	0.54520774	0.5453188	0.5441552

mean_per_class_error	0.4533808	0.0016683937	0.45232338	0.44926223	0.45479223	0.4546812	0.45584485
mse	0.31210867	8.8877673E-4	0.31267777	0.31123692	0.31152028	0.3108118	0.31429666
r2	0.8732805	5.2272924E-4	0.8727144	0.8735932	0.8737134	0.8742182	0.8721636
rmse	0.55866575	7.94834E-4	0.55917597	0.5578861	0.55814	0.55750495	0.5606217

Test Set Metrics:

MSE: 0.1151542
RMSE: 0.3393438
Logloss: 0.3868228
Mean Per-Class Error: 0.07395107
Confusion Matrix:
Confusion Matrix: Row labels: Actual class; Column labels: Predicted class

	-2	-1	0	1	2	Error	Rate
-2	35337	159	6	56	140	0.0101 =	361 / 35,698
-1	3309	18031	28	70	72	0.1617 =	3,479 / 21,510
0	403	32	17962	33	286	0.0403 =	754 / 18,716
1	298	23	37	18700	2873	0.1473 =	3,231 / 21,931
2	200	29	8	130	35292	0.0103 =	367 / 35,659
Totals	39547	18274	18041	18989	38663	0.0614 =	8,192 / 133,514

Hit Ratio Table:
Top-5 Hit Ratios:

	k	hit_ratio
1	1	0.938643
2	2	0.997004
3	3	0.999970
4	4	1.000000
5	5	1.000000

Table S3e. Model fitting output for the meta-learner "Ensemble"

H2OMultinomialModel: stackedensemble
Number of Base Models: 3
Base Models (count by algorithm type): drf gbm glm

Metalearner algorithm: glm
Metalearner cross-validation fold assignment:
Fold assignment scheme: Modulo
Number of folds: 5

Training Set Metrics:

MSE: 0.1039267

RMSE: 0.3223766
Logloss: 0.3606753
Mean Per-Class Error: 0.08435653

Confusion Matrix: Row labels: Actual class; Column labels: Predicted class

	−2	−1	0	1	2	Error	Rate
−2	34764	442	173	181	138	0.0262 =	934 / 35,698
−1	2110	18990	244	91	75	0.1172 =	2,520 / 21,510
0	239	77	18208	158	34	0.0271 =	508 / 18,716
1	454	59	241	17137	4040	0.2186 =	4,794 / 21,931
2	264	45	125	733	34492	0.0327 =	1,167 / 35,659
Totals	37831	19613	18991	18300	38779	0.0743 =	9,923 / 133,514

Hit Ratio Table:
Top-5 Hit Ratios:

	k	hit_ratio
1	1	0.925678
2	2	0.987949
3	3	0.995536
4	4	0.999049
5	5	1.000000

Validation Set Metrics:

MSE: 0.1039267
RMSE: 0.3223766
Logloss: 0.3606753
Mean Per-Class Error: 0.08435653

Confusion Matrix: Row labels: Actual class; Column labels: Predicted class

	−2	−1	0	1	2	Error	Rate
−2	34764	442	173	181	138	0.0262 =	934 / 35,698
−1	2110	18990	244	91	75	0.1172 =	2,520 / 21,510
0	239	77	18208	158	34	0.0271 =	508 / 18,716
1	454	59	241	17137	4040	0.2186 =	4,794 / 21,931
2	264	45	125	733	34492	0.0327 =	1,167 / 35,659
Totals	37831	19613	18991	18300	38779	0.0743 =	9,923 / 133,514

Hit Ratio Table:
Top-5 Hit Ratios:

	k	hit_ratio
1	1	0.925678
2	2	0.987949
3	3	0.995536
4	4	0.999049
5	5	1.000000

Cross-Validation Set Metrics:

MSE: 0.2905095
RMSE: 0.5389893
Logloss: (0.8525439
Mean Per-Class Error: 0.4090067

Top-5 Hit Ratios:

	k	hit_ratio
1	1	0.640030
2	2	0.893577
3	3	0.963120
4	4	0.986556
5	5	1.000000

Test Set Metrics:

MSE: 0.1039267
RMSE: 0.3223766
Logloss: 0.3606753
Mean Per-Class Error: 0.08435653

Confusion Matrix: Row labels: Actual class; Column labels: Predicted class

	−2	−1	0	1	2	Error	Rate
−2	34764	442	173	181	138	0.0262 =	934 / 35,698
−1	2110	18990	244	91	75	0.1172 =	2,520 / 21,510
0	239	77	18208	158	34	0.0271 =	508 / 18,716
1	454	59	241	17137	4040	0.2186 =	4,794 / 21,931
2	264	45	125	733	34492	0.0327 =	1,167 / 35,659
Totals	37831	19613	18991	18300	38779	0.0743 =	9,923 / 133,514

Hit Ratio Table:
Top-5 Hit Ratios:

	k	hit_ratio
1	1	0.925678
2	2	0.987949
3	3	0.995536
4	4	0.999049
5	5	1.000000

Table S4. Proportions of cVEP classes of the nsSNVs grouped by the chromosomes.

Chr	Pathogenic	Likely_pathogenic	Uncertain	Likely_benign	Benign

1	0.170	0.043	0.252	0.374	0.162
2	0.171	0.051	0.308	0.350	0.120
3	0.186	0.049	0.273	0.381	0.111
4	0.177	0.039	0.256	0.389	0.138
5	0.185	0.046	0.273	0.377	0.120
6	0.170	0.039	0.241	0.392	0.159
7	0.170	0.041	0.248	0.373	0.169
8	0.175	0.041	0.283	0.367	0.133
9	0.166	0.046	0.254	0.369	0.166
10	0.179	0.043	0.263	0.370	0.145
11	0.163	0.042	0.252	0.388	0.155
12	0.174	0.056	0.278	0.381	0.112
13	0.175	0.047	0.302	0.341	0.136
14	0.162	0.056	0.265	0.386	0.132
15	0.175	0.047	0.271	0.361	0.146
16	0.168	0.043	0.260	0.373	0.156
17	0.181	0.054	0.271	0.355	0.138
18	0.178	0.041	0.258	0.388	0.135
19	0.136	0.034	0.189	0.418	0.223
20	0.183	0.049	0.274	0.375	0.119
21	0.169	0.036	0.260	0.385	0.150
22	0.178	0.044	0.261	0.381	0.136
X	0.188	0.061	0.183	0.391	0.177
Y	0.070	0.001	0.018	0.330	0.581
M	0.404	0.000	0.000	0.042	0.553
All chromosomes	0.172	0.046	0.258	0.377	0.148
gnomAD,“control”	0.100	0.020	0.237	0.433	0.211

Table S5. Improvement in accuracy of cVEP predictions by including *indispensability* (*i*-score) for essential half of genes for *“ClinVar”* dataset.

Gene group	*i* -score* range	*i*-score included	AUC	FP %*	FN %*
Essential	0.5 - 1.0	**No**	0.986	**4.9**	**6.1**
Essential	0.5 - 1.0	**Yes**	0.994	**2.1**	**2.0**

*The columns: Gene group, indispensability score (i-score), AUC, False-Positive/-Negative Errors
